# Competition between homologous chromosomal DNA and exogenous donor DNA to repair CRISPR/Cas9-induced double-strand breaks in *Aspergillus  niger*

**DOI:** 10.1186/s40694-024-00184-3

**Published:** 2024-10-15

**Authors:** Selina Forrer, Mark Arentshorst, Prajeesh Koolth Valappil, Jaap Visser, Arthur F. J. Ram

**Affiliations:** Institute Biology Leiden, Microbial Sciences, Fungal Genetics and Biotechnology, Leiden University, Sylviusweg 72, Leiden, 2333 BE The Netherlands

**Keywords:** *Aspergillus niger*, CRISPR/Cas9 genome editing, Cell factory, Multicopy strains, DNA repair, Protein production

## Abstract

**Background:**

*Aspergillus niger* is well-known for its high protein secretion capacity and therefore an important cell factory for homologous and heterologous protein production. The use of a strong promoter and multiple gene copies are commonly used strategies to increase the gene expression and protein production of the gene of interest (GOI). We recently presented a two-step CRISPR/Cas9-mediated approach in which glucoamylase (*glaA*) landing sites (GLSs) are introduced at predetermined sites in the genome (step 1), which are subsequently filled with copies of the GOI (step 2) to achieve high expression of the GOI.

**Results:**

Here we show that in a *ku70* defective *A. niger* strain (*Δku70*), thereby excluding non-homologous end joining (NHEJ) as a mechanism to repair double-stranded DNA breaks (DSBs), the chromosomal *glaA* locus or homologous GLSs can be used to repair Cas9-induced DSBs, thereby competing with the integration of the donor DNA containing the GOI. In the absence of exogenously added donor DNA, the DSBs are repaired with homologous chromosomal DNA located on other chromosomes (inter-chromosomal repair) or, with higher efficiency, by a homologous DNA fragment located on the same chromosome (intra-chromosomal repair). Single copy inter-chromosomal homology-based DNA repair was found to occur in 13–20% of the transformants while 80–87% of the transformants were repaired by exogenously added donor DNA. The efficiency of chromosomal repair was dependent on the copy number of the potential donor DNA sequences in the genome. The presence of five homologous DNA sequences, resulted in an increased number (35–61%) of the transformants repaired by chromosomal DNA. The efficiency of intra-chromosomal homology based DSB repair in the absence of donor DNA was found to be highly preferred (85–90%) over inter-chromosomal repair. Intra-chromosomal repair was also found to be the preferred way of DNA repair in the presence of donor DNA and was found to be locus-dependent.

**Conclusion:**

The awareness that homologous chromosomal DNA repair can compete with donor DNA to repair DSB and thereby affecting the efficiency of multicopy strain construction using CRISPR/Cas9-mediated genome editing is an important consideration to take into account in industrial strain design.

**Supplementary Information:**

The online version contains supplementary material available at 10.1186/s40694-024-00184-3.

## Introduction

The filamentous fungus *Aspergillus niger* is one of the important cell factories used in industry for the production of organic acids, proteins, enzymes, and secondary metabolites (Behera 2020; Li et al. 2020; Cairns et al. 2018, 2021; Yu et al. 2021). Contributing to its success as a cell factory has been the early availability of full genome sequences, the development of -omics technologies and efficient genome editing systems (reviewed by Cairns et al. 2021). The implementation of non-homologous end joining mutants marks a first major step in facilitating and improving the efficiency in the generation of gene deletion mutants (Meyer et al. 2007; Carvalho et al. 2010) or other types of targeted genome editing (Arentshorst et al. 2015). The second major step has been the implementation of CRISPR/Cas9 as a genome editing tool. Since its introduction in 2015 (Nødvig et al. 2015), CRISPR/Cas9-based genome editing has become the standard genome editing tool to perform *A. niger* gene modifications (see for review Jin et al. 2022).

The power of CRISPR/Cas9 genome editing is based on the ability of the Cas9 nuclease to create a DNA double-strand break (DSB) at a predetermined site in the genome. The predetermined site is selected by designing a single guide RNA (sgRNA) that consists of a CRISPR RNA (crRNA) harboring a 20-nucleotide long target specific DNA sequence at the 5ʹ-end, and a transactivating crRNA (tracrRNA) at the 3ʹ end for Cas9 binding. The requirement for the design of the crRNA is the presence of a Protospacer Adjacent Motif (PAM) adjacent to the binding site of the guiding crRNA at the target site (Garneau et al. 2010). The crRNA part guides the sgRNA/Cas9 complex to the target site where the Cas9 endonuclease will generate a DSB three nucleotides upstream of the PAM sequence (Sander and Joung 2014). DSBs are extremely deleterious for the cell and need to be repaired to prevent chromosome loss. There are two major pathways in fungi to repair DSBs in DNA, called the non-homologous end joining (NHEJ) pathway and the homologous recombination (HR) pathway. In the NHEJ pathway the Ku70/Ku80 proteins bind to the DSB ends and recruit other factors (e.g. Lig4) to ligate the two broken DNA ends together. The NHEJ pathway is error prone and often causes permanent alterations. In fungal CRISPR/Cas9 genome editing in which the CRISPR/Cas9 plasmid-based approach is used, DSB repairs without alterations will be subjected to a second round of Cas9 nuclease activity until the target site is damaged. The HR pathway, which involves among others Rad51 and Rad52 proteins, is a relatively accurate repair process dependent on the guidance of homologous DNA (donor DNA or repair DNA) that is used to repair the DSB by homologous recombination. The repair DNA in CRISPR/Cas9-based genome editing of fungi normally consists of flanking regions from both sites adjacent to the DSB to make specific knockouts (van Leeuwe et al. 2019) or knockins (Kun et al. 2020). For gene editing studies in filamentous fungi the preferred and most often used method is to add plasmid or PCR-derived repair DNA during the transformation. This repair DNA is also taken up during transformation and is used as a template for DNA repair.

Song et al. (2018) have shown that for *A. niger* the use of NHEJ mutants in CRISPR/Cas9-mediated genome editing has major advantages over the use of wild type strains. By taking advantage of NHEJ mutants, high efficiencies of targeted integration are obtained (Song et al. 2018). The same study also showed that a NHEJ-deficient mutant can also be used to test the efficacy of the sgRNA. The absence of donor DNA during a transformation with an efficient sgRNA in a NHEJ-deficient mutant is expected to yield no transformants. In addition, constructing mutants in a NHEJ background has the advantage that off-target mutations are drastically reduced (Garrigues et al. 2023).

The possibilities of CRISPR/Cas9-mediated genome editing have opened new strategies in terms of *A. niger* strain construction. Part of that is due to the fact that the editing method can be conducted “marker free”. This is accomplished by expressing the Cas9 gene and the sgRNA from an autonomously replicating plasmid, the AMA vector (Aleksenko and Clutterbuck 1996). The AMA element on the plasmid prevents integration and supports replication. With the loss of selection pressure, the AMA-containing plasmid is easily lost during the following conidiation cycle which allows iterative gene editing cycles (Nødvig et al. 2015; van Leeuwe et al. 2019). By performing iterative cycles, strains carrying seven (van Leeuwe et al. 2019) or seventeen gene deletions (Arentshorst et al. 2021) have been generated. In each cycle up to three genes can be deleted. Higher number of gene deletions at the same time reduce the number of transformants obtained (van Leeuwe et al. 2019). The iterative gene editing option also allows to perform efficient gene complementation studies (Seekles et al. 2023).

The CRISPR/Cas9 technology in combination with the use of a NHEJ-deficient mutant was also used to create an *A*. *niger* expression host for protein production. The use of NHEJ mutants is fundamental for the approach to construct production strains carrying multiple copies of the gene of interest. In a *ku70-* (*kusA*) deficient strain ten loci were deleted via CRISPR/Cas9 genome editing and the coding regions of these genes were replaced by a so-called glucoamylase landing site (GLS). Each landing site consists of the *glaA* promoter region (*PglaA*) and the *glaA* terminator region (*TglaA*) and in between these regions one or more unique DNA sequences (dubbed KORE sequences) were introduced for which unique Cas9-compatible sgRNAs were designed (Arentshorst et al. 2023). The genes selected for deletion were genes encoding abundantly present extracellular enzymes and included glucoamylase (*glaA*), amylase (*aamA*) and alpha-glucosidase (*agdA*), four aspartic proteases (*pepA*,* pepB*,* pepN and NRRL3_10267)*, a carboxypeptidase (NRRL3_06629), glucose oxidase (*goxC*) and oxaloacetate hydrolase (*oahA*) involved in medium acidification. Together with the deletion of alpha-glucosidase (*agdA)*, two neighboring genes *amyA* and *prtT* encoding an amylase and a transcription factor involved in protease induction respectively, were also deleted and replaced by a single landing site. The expression host constructed contains ten landing sites (in total 12 genes deleted) which can be filled by a gene or genes of interest by creating a DSB using a specific KORE directed sgRNA in combination with repair DNA consisting of the gene(s) of interest which are flanked to *PglaA* and *TglaA* regions (Arentshorst et al. 2023). Note that the use of a NHEJ-deficient mutant is essential, otherwise the Cas9-induced DSBs will be repaired by non-homologous end joining and via homologous recombination and integration of the GOI. Since not all 10 landing sites can be filled simultaneously, the filling takes place in steps in which particular KORE sites are targeted by KORE-specific sgRNAs to create DSBs. We noticed in carrying out experiments to fill the landing sites with GOIs, that not all landing sites were filled. To understand why not all Cas9 targeted landing sites were repaired via incorporation of the GOI, a specific set of *A. niger* strains was generated to study this phenomenon.

In this paper we show that in addition to the exogenously added repair DNA containing the GOI, the DSB can also be repaired by homologous landing sites present is the genome that were not targeted by Cas9. We also show that intra-chromosomal repair by a homologous landing site that is in close proximity (46 kb) to the introduced DSB is preferred over inter-chromosomal repair. Our findings are important in designing strain engineering strategies to optimize fungi as host for protein production.

## Materials and methods

### Strains and growth conditions

The *A. niger* strains used in this study are listed in Table 1. All strains are derived from the N400 (NRRL3) *A. niger* wild type strain (Bos et al. 1988; Demirci et al. 2021). A *∆ku70* derivate strain in N400 was previously constructed and used in this study (Demirci et al. 2021). Strains were grown in liquid or solidified (containing 1.5% (w/v) Scharlau agar) minimal medium (MM) or in complete medium (CM) as described (Arentshorst et al. 2012). *Escherichia coli* DH5α was used for plasmid construction and cultured at 37 °C in Luria-Bertani medium, with ampicillin (100 µg/mL).

**Table 1:** Strains used in this study

Strain name	Genotype	References
N400	wild-type NRRL3; ATCC9092; CBS120.49	Bos et al. (1988)
MA612.27	*kusA::DR-amdS-DR* in N400	Demirci et al. (2021)
MA950.1	*∆glaA::PglaA-KORE1-TglaA* in MA612.27	Arentshorst et al. (2023)
MA952.1	*∆aamA::PglaA-KORE1-TglaA* in MA950.1	Arentshorst et al. (2023)
PKV1.7	*∆amyA-agdA::PglaA-KORE1-TglaA* in MA952.1	This study
PKV2.5	*∆agdB::PglaA-KORE4-TglaA* in PKV7.1	This study
PKV3.1	*∆pepA::PglaA-KORE4-TglaA* in PKV2.5	This study
PKV7.1	*∆pepB::PglaA-KORE5-TglaA* in PKV3.1	This study
SF11.1	*∆glaA::PglaA-glaA538-His-TglaA* *∆aamA::PglaA-glaA538-His-TglaA* *∆amyA-agdA::PglaA-gla538-His-TglaA* *∆agdB::PglaA-glaA538-his-TglaA* *∆pepA::PglaA-glaA538-TglaA* *∆pepB::PglaA-KORE5-TglaA*	This study
MA1025.4	*∆glaA::PglaA-KORE1-TglaA* *∆aamA::PglaA-KORE1-TglaA* *∆amyA-agdA-prtT::PglaA-KORE1-TglaA* *∆pepA::PglaA-KORE1-TglaA* *∆pepB::PglaA-KORE4-TglaA* *∆pepN::PglaA-KORE5-TglaA*	Arentshorst et al. (2023)
PKV5.4	*∆pepB::PglaA-KORE5-TglaA* in MA612.27	This study
SF29.1	*∆glaA* in PKV5.4	This study
SF30 series	pFC332-KORE5 no donor DNA transformants in PKV5.4	This study
SF32 series	pFC332-KORE5 2 µg *PglaA-lux-TglaA* donor DNA transformants in PKV5.4	This study
SF33 series	pFC332-KORE5 12 µg *PglaA-lux-TglaA* donor DNA transformants in PKV5.4	This study
SF34 series	pFC332-KORE5 no donor DNA transformants in SF11.1	This study
SF35 series	pFC332-KORE5 2 µg *PglaA-lux-TglaA* donor DNA transformants in SF11.1	This study
SF36 series	pFC332-KORE5 12 µg *PglaA-lux-TglaA* donor DNA transformants in SF11.1	This study
MA1125.1	pFC332-KORE5 no donor DNA transformant in SF29.1	This study
MA1124 series	pFC332-KORE5 2 µg *PglaA-lux-TglaA* donor DNA transformants in SF29.1	This study
MA1120 series	pFC332-KORE4 no donor DNA in MA1025.4	This study
MA1121 series MA1122 series MA1123 series	pFC332-KORE4 2 µg *PglaA-lux-TglaA* donor DNA transformants in MA1025.4 pFC332-KORE5 no donor DNA in MA1025.4 pFC332-KORE5 2 µg *PglaA-lux-TglaA* donor DNA transformants in MA1025.4	This study This study This study

### CRISPR/Cas9 plasmid constructions

All *A. niger* transformations in this study were performed by using the pFC332-based marker-free CRISPR/Cas9 genome editing method (Nødvig et al. 2015; van Leeuwe et al. 2019) or by cloning of crRNAs into plasmid pLML001, derived from pFC332 (Arentshorst et al. 2023). The vector pLML001 supports a one-step sgRNA targeting sequence incorporation into the vector and utilizes the Pro1 tRNA RNA polymerase III promoter for sgRNA transcription (Song et al. 2018; Arentshorst et al. 2023). sgRNA targets were designed using the CHOPCHOP web-tool (Labun et al. 2016) or the CRISPR gRNA (guide RNA) Design Tool for Eukaryotic Pathogens (uga.edu) available at http://grna.ctegd.uga.edu/. Cloning of guide RNA sequences in either pFC332 or pLML001 were performed as described previously (van Leeuwe et al. 2019; Arentshorst et al. 2023). All primers used in this study are listed in Supplementary Table 1.

### Construction of donor DNA fragments

**Fig. 1:**
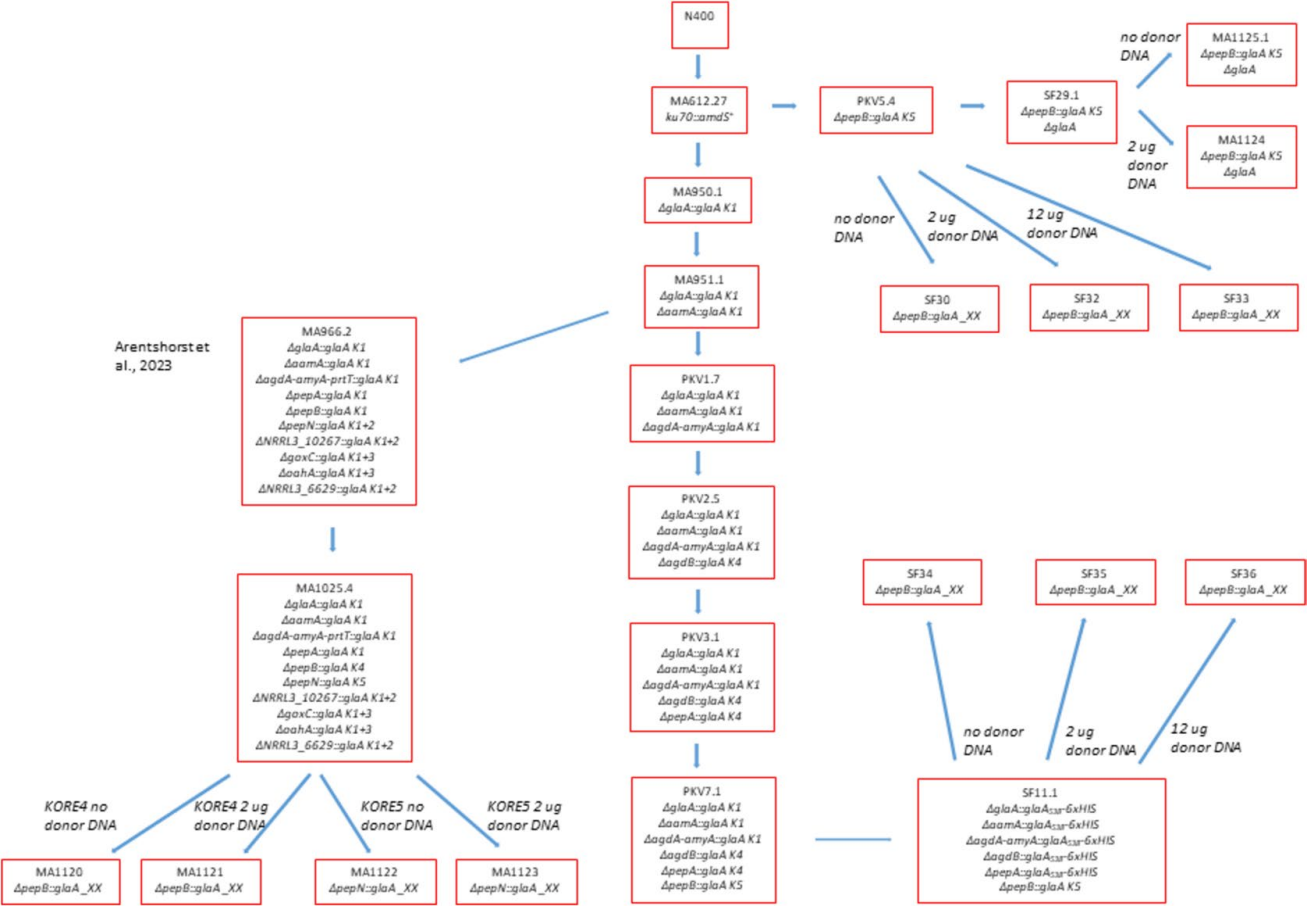
Schematic overview of the lineage of *A. niger* strains and predecessor strains starting with wild-type strain N400 used in this study

Donor DNA fragments to delete target genes and at the same time integrate a glucoamylase landing site (GLS), were amplified by fusion PCR using a set of gene specific primers (Supplementary Table 1) as described previously (Arentshorst et al. 2023). Iterative transformations were performed to finally obtain PKV7.1. This strain has six gene loci deleted and replaced by a GLS. Please note that *agdA* and *amyA* are neighboring genes and that both genes were deleted and replaced by a single GLS. The entire strain lineage is shown in Fig. 1. After each transformation round, candidate deletion strains were purified and grown without hygromycin to allow plasmid loss. Genomic DNA of hygromycin sensitive strains was isolated and used in a diagnostic PCR to confirm proper deletion of the target gene and integration of the landing site.

Two donor DNA fragments (*PglaA-glaA*_*538*_::*6xHis-TglaA* and *PglaA-lux-TglaA*) to insert His-tagged glucoamylase or the luciferase reporter gene at glucoamylase landing sites respectively, were amplified by fusion PCR using primers listed in Supplementary Table 1. The final PCR fragment contains the *PglaA* promoter (634 bp), either the *glaA*_*538*_::*6xHis* gene or the luciferase gene (*lux*) and the *TglaA* terminator sequence (547 bp). Fusion PCR fragments were cloned in pJet1.2 to give pMA_ *glaA*_*538*_::*6xHis* and pPKV_*PglaA-lux*, respectively. Both fusion constructs were sequenced to confirm that no PCR errors were introduced. DNA fragments containing the *PglaA-glaA::6xHis-TglaA or PglaA-lux-TglaA* expression cassette were excised from their respective plasmids with *Pme*I and subsequently purified from agarose gel and used for transformation. Successful integration of the gene of interest at each locus was verified by performing a diagnostic PCR on genomic DNA of transformants using locus specific primers (Supplementary Table 1).

The DNA fragment used to remove the *glaA* locus, including 664 nucleotides upstream of the *glaA* encoding gene and 572 nucleotides downstream of the *glaA* gene, was also generated by fusion PCR using primers listed in Supplementary Table 1. The fusion fragment of the predicted size (1417 bp) was purified from agarose gel and used for transformation of PKV5.4. Successful deletion of the *glaA* locus in PKV5.4 was verified by diagnostic PCR. Strain SF29.1 was used to perform subsequent studies.

### Transformation of *A.niger* and analysis of the transformants

*A. niger* transformants were obtained by selection for hygromycin resistance as described (Arentshorst et al. 2012) using a final concentration of 100 µg/mL hygromycin. Transformation of the recipient strain were done using the pFC332-sgRNA plasmid or pLML001-sgRNA plasmid-based method (both 2 µg DNA) together with the repair DNA fragment (2–12 µg DNA) unless stated differently. Primary transformants were purified on MM supplemented with hygromycin, followed by a purification step on hygromycin free MM plates to allow loss of the AMA1-containing plasmids. Afterwards, the loss of these plasmids was confirmed by growth analyses on MM supplemented with hygromycin. Correct integration of the donor DNA was verified by isolating genomic DNA as described (Arentshorst et al. 2012) and subsequent diagnostic PCRs.

## Results and discussion

### Construction and analysis of *A.niger* strains to study CRISPR/Cas9-induced double-strand break repair

**Fig. 2:**
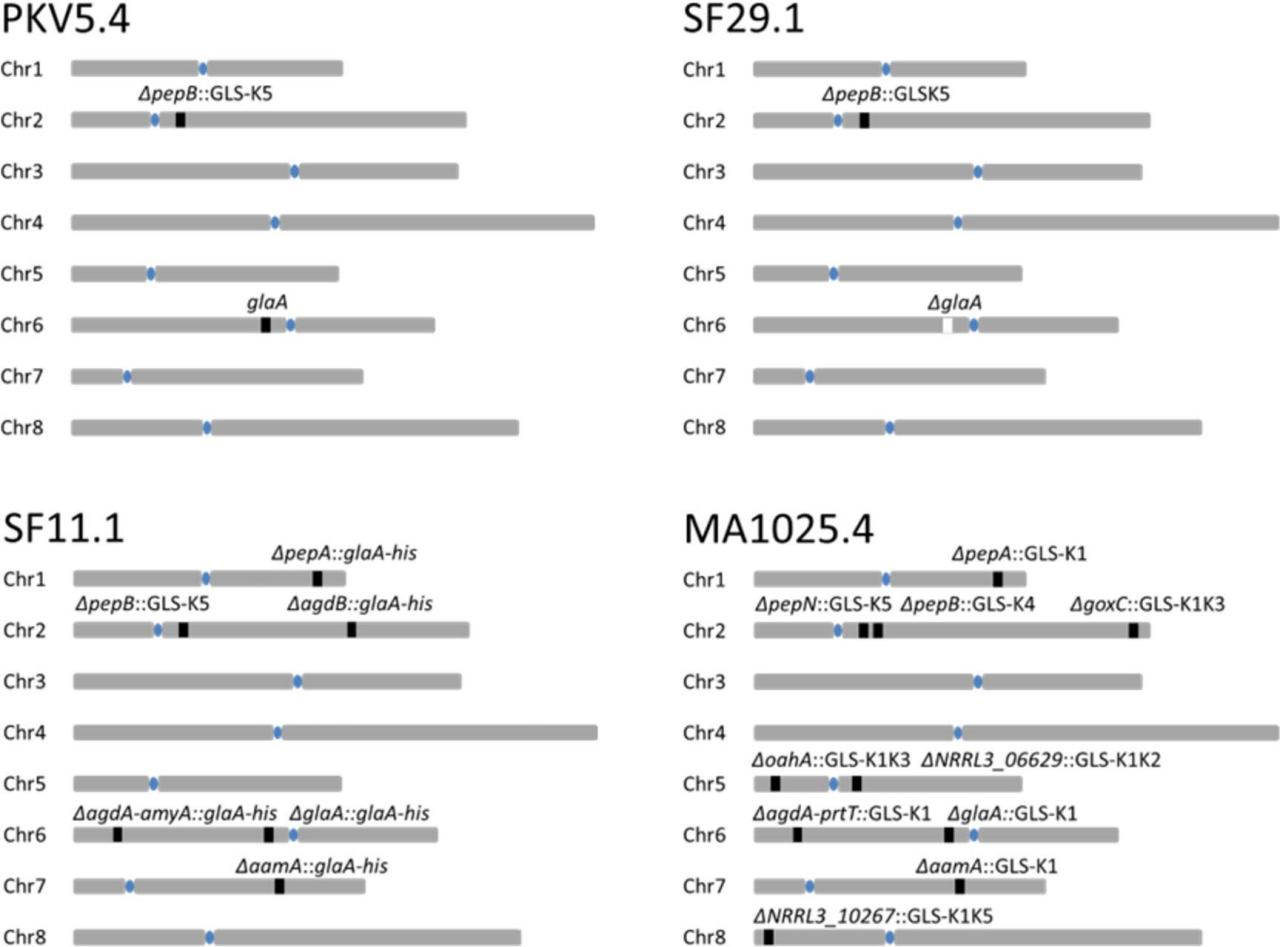
Schematic representation of the chromosomal location of glucoamylase (*glaA*) and glucoamylase landing sites (GLS) at specific gene loci in various *A. niger* strains. For each locus the respective KORE site in the GLS is shown, or it is indicated that the GLS is filled with the *gla538-His* gene. Centromeres are indicated with a blue ovals

For the initial assessment to determine the efficiency of chromosomal versus exogenously added donor DNA to repair Cas9-induced DSBs, three different *A. niger* strains (PKV5.4, SF29.1 and SF11.1) were constructed. All three strains are derived from the *ku70* deletion strain MA612.27 (Demirci et al. 2021). The strain lineage of these strains is shown in Fig. 1 and the genotypes of the strains are listed in Table 1. In the first strain (PKV5.4), the *pepB* gene was deleted and the *pepB* coding region was replaced by a glucoamylase landing site (GLS) (Arentshorst et al. 2023). As schematically shown in Fig. 2A, the *pepB* gene is located at the right arm of chromosome 2. The landing site consists of part of the glucoamylase promoter (*PglaA)* region (− 634 to − 247 bp upstream of ATG) and the glucoamylase terminator (*TglaA*) region (from + 142 to + 547 bp after the stop codon). In the case of the GLS at the *pepB* locus in PKV5.4, the *PglaA* and *TglaA* regions are separated by a unique DNA sequence (KORE5 sequence) for which a specific sgRNA has been designed. The KORE5 sequence is based on a *brnA* specific sgRNA, in which the 20 bp-long target-complementary CRISPR RNA (crRNA) sequence has been changed to make the sgRNA specific and unique in the *A. niger* genome (Arentshorst et al. 2023). A second strain (SF29.1) is a direct derivative of PKV5.4 in which, in addition to the *pepB::PglaA-KORE5-TglaA* deletion/insertion, also the entire *glaA* locus was deleted. The *glaA* gene is localized on the left arm of chromosome 6 (Fig. 2B). Not only the *glaA* coding region is deleted but also part of the promoter region (− 664 bp before the start codon) and the terminator region (+ 572 bp after the stop codon) (Fig. 2B).

As a starting strain to construct the third strain used in this part of the study (SF11.1) *A. niger* strain PKV7.1 was constructed (Fig. 1). PKV7.1 has deletions in the glucoamylase gene (*glaA*), the acid amylase gene (*aamA*), the clustered α-glucosidase/amylase (*agdA/amyA)* genes, the α-glucosidase (*agdB)* gene, and in two genes encoding aspartic proteases (*pepA* and *pepB*) (Fig. 2C). In all cases, the coding region of the genes was replaced by a GLS (*PglaA-KORE-TglaA*). For the deletion of the *glaA*, *aamA* and *agdA/amy*A loci, a KORE1 sequence was inserted between the *PglaA* and *TglaA* sequences, for *agdB* and *pepA* the KORE4 and for the *pepB* locus the KORE5 sequence was used. All loci of the resulting strain PKV7.1 that was constructed by iterative CRISPR/Cas9-based transformation cycles, were PCR amplified and sequenced to confirm proper deletion and integration of the landing sites (Fig. 1 and data not shown). Next, the landing sites introduced on the original *glaA*,* aamA*,* agdA/amyA*,* agdB* and *pepA* loci were filled by one round of CRISPR/Cas9-mediated transformation using the *PglaA-glaA*_*503*_::*6xHis-TglaA* fusion construct as donor DNA and using KORE1-(*glaA*,* aamA*,* agdA/amyA*) and KORE4-(*agdB* and *pepA*) specific guides. Transformant SF11.1 was selected as diagnostic PCRs confirmed that in this strain all five landing sites at the *glaA*,* aamA*,* agdA/amyA*,* agdB*, and *pepA* loci were filled by the donor DNA construct (data not shown). Note that the *PglaA-KORE5-TglaA* landing site at the *pepB* locus is still present in SF11.1 (Fig. 2C).

### The occurrence of inter-chromosomal DNA repair of CRISPR/Cas9-induced DSB in *A.niger*

To test whether a CRISPR/Cas9-induced DSB could be repaired by a homologous DNA sequence located on another chromosome (inter-chromosomal repair), strain PKV5.4 was transformed with a CRISPR/Cas9 autonomously replicating plasmid, also containing a sgRNA specific for a KORE5 sequence (pFC332_sgKORE5). This KORE5 sequence is part of a so-called glucoamylase landing site (GLS) which was used to delete the *pepB* gene and to create a GLS on the *pepB* locus (see Arentshorst et al. 2023 for details). Because in strain PKV5.4 the *ku70* gene is disrupted, no NHEJ can occur and the DSB repair depends therefore on homologous recombination. Without the addition of exogenous donor DNA, the DSB in PKV5.4 can in theory only be repaired by the endogenous *glaA* locus. The *pepB* gene (NRRL3_01627) and *glaA* gene (NRRL3_08300) are localized on the right arm of chromosome 2 and the left arm of chromosome 6, respectively (Fig. 2A). Transformation of PKV5.4 with plasmid pFC332-sgKORE5 without donor DNA resulted in strong reduction in the number of transformants (about 85% reduction compared to a positive control transformation with pFC332) (Supplemental Fig. 1 and Supplemental Table 2). Nineteen transformants were purified and subjected to one round of non-selective growth to allow plasmid loss. Genomic DNA of transformants that lost the Cas9/sgRNA plasmid was isolated and the *pepB* locus was analyzed by diagnostic PCR using *pepB* specific primers to analyze the insertion event. Repair of the DSB by the endogenous *glaA* locus is expected to result in a PCR fragment of 3846 bp. As shown in Supplemental Fig. 2A and summarized in Table 2, the sgKORE5 sgRNA induced DSB in all 19 transformants was repaired by the endogenous *glaA* locus based on the size of the PCR fragment and DNA sequencing of one of the PCR fragments. This shows that a CRISPR/Cas9-induced DSB can be repaired by homologous DNA present on other chromosomes.

**Table 2:** Homologous recombination frequencies after Cas9-induced DSB using KORE5 specific guide RNA

Strain	Donor DNA	# of transformants analysed	Chromo- somal repair	Donor DNA repair	PCR product aberrant size	No PCR product	Chromo-somal repair %	Donor DNA repair %
SF29.1	−	1*	−	−	−	−	−	−
SF29.1	2 µg *PglaA-lux-TglaA*	10	−	10	−	−	−	100%
PKV5.4	−	20	19	−	1	−	95%	−
PKV5.4	2 µg *PglaA-lux-TglaA*	16	2	14	−	−	13%	87%
PKV5.4	12 µg *PglaA-lux-TglaA*	20	3	17	−	−	15%	85%
SF11.1	−	20 (19**)	19	−	−	1	100%	−
SF11.1	2 µg *PglaA-lux-TglaA*	20 (18**)	7	11	−	2	35%	55%
SF11.1	12 µg *PglaA-lux-TglaA*	18 (16**)	11	5	−	2	61%	29%

* PCR product is identical to orginal GLS, indicating no CAS9 activity in the single transformant obtained; ** actual number of transformants with a conclusive PCR result

In strain SF29.1 the entire endogenous *glaA* locus, including 664 bp promoter region and 572 bp terminator region of the *glaA* gene, was deleted. The removal of the entire *glaA* locus, including the flanking regions, resulted in a strong reduction in the number of colonies after a transformation using a KORE5 guide to introduce a DSB at the GLS-KORE5 site at the *pepB* locus. Only a single transformant was obtained (Supplemental Fig. 1 and Supplemental Table 2). A diagnostic PCR on genomic DNA and subsequent sequencing of the PCR product of this transformant showed that the GLS-KORE5 was unaltered indicating that this transformant escaped somehow CRISPR editing. The experiments in PKV5.4 and in SF29.1 clearly show that the endogenous *glaA* locus can be used to repair the KORE5 induced DSB at the GLS at the *pepB* locus.

Inter-chromosomal DNA repair was also assessed in *A. niger* strain SF11.1. This strain also has a KORE5-containing GLS at the *pepB* locus and has five potential homologous donor sequences (*Pgla-GlaA*_538_::*6xHis-TglaA* gene copies) at five chromosomal locations. These loci include *glaA* (NRRL3_08300, Chr 6), *aamA* (NRRL3_09875, Chr 7), *agdA/amyA* (NRRL3_07700/07699, Chr 6), *agdB* (NRRL3_02524, chr 2) and *pepA* (NRRL3_00987, Chr 1) (Fig. 2C). Transformation of SF11.1 with plasmid pFC332-sgKORE5 without the addition of donor DNA resulted also in strong reduction in the number of transformants (about 85% reduction compared to a positive control transformation with pFC332) (Supplemental Fig. 1 and Supplemental Table 2). Again twenty transformants were purified and subjected to plasmid loss and analyzed. In all 19 strains with a successful PCR, the diagnostic PCR indicated that the DSB break at the *pepB* locus was repaired by one of the *Pgla-GlaA*_*538*_::*6xHis-TglaA* chromosomal loci (Table 2 and Supplemental Fig. 3A). Because all *Pgla-GlaA*_*538*_::*6xHis-TglaA* copies are identical it is not possible to identify which copy was used for repair. Again this result shows that a CRISPR/Cas9-induced DSB can be repaired by homologous chromosomal DNA.

### Comparison of the efficiency of inter-chromosomal homology-based repair versus exogenously added donor DNA repair

To compare the preference of DSB repair by inter-chromosomal homologous donor sequences or by exogenously added donor DNA, strains PKV5.4 and SF11.1 were transformed with plasmid pFC332-sgKORE5 with the addition of donor DNA. Since this set of transformations was performed using the same batch of protoplasts as before (no donor DNA transformation), transformation efficiencies can be compared between these experiments. The donor DNA used is a 2828 bp *PglaA-lux-TglaA* PCR fragment consisting of 635 bp of *PglaA*, the *lux* gene (1647 bp) and the *TglaA* region (547 bp). To transform each strain, two different amounts of PCR fragments (2–12 µg) were used. The addition of donor DNA stimulated transformation frequencies significantly compared to the no DNA transformations and the numbers of transformants obtained reached efficiencies between 50 and over 100% compared to the positive control (2 µg pFC332) (Supplemental Fig. 1). From each transformation condition, twenty transformants were purified, subjected to plasmid loss and plasmid-free transformants were analyzed by diagnostic PCR. Integration of the exogenously added donor DNA (*PglaA-lux-TglaA*) is expected to result in a PCR fragment of 3321 bp. Repair by the endogenous *glaA* gene in PKV5.4 will result in a PCR fragment of 3846 bp, while repair by an endogenous *Pgla-GlaA*_*538*_::*6xHis-TglaA* copy in SF11.1 is expected to result in a PCR fragment of 3558 bp. The different sizes of the PCR fragments enables to conclude by which donor DNA the KORE5 mediated DSB at the *pepB* locus was repaired.

Transformation of PKV5.4 with pFC332-sgKORE5 with donor DNA (2–12 µg) increased transformation efficiencies compared to transformation without donor DNA. This indicated that the donor DNA was used preferentially to repair the DSB (Supplemental Fig. 1, Supplemental Table 2). Genomic DNA of sixteen transformants obtained by including 2 µg donor DNA and of twenty transformants obtained by including 12 µg donor DNA, was isolated and the *pepB* locus was analyzed by diagnostic PCR. For both the 2 µg and 12 µg donor DNA transformation experiments, the majority of the transformants (14/16; 87% and 16/20; 80%, respectively) were repaired by the exogenously added donor DNA (Table 2 and Supplemental Fig. 2B and 2 C). Apparently, the higher amount of donor DNA did not have an impact on the efficiency of donor DNA-mediated repair.

Similarly, SF11.1 was transformed with pFC332-sgKORE5 with donor DNA (2–12 µg). Again, the addition of donor DNA increased transformation efficiency compared to a transformation without donor DNA, indicating that the donor DNA was used for repairing the DSB (Supplemental Fig. 1). Analysis of eighteen transformants obtained with the 2 µg donor DNA revealed that eleven were repaired by donor DNA (11/18; 61%) and analysis of sixteen transformants obtained with the 12 µg donor DNA transformation indicated that five transformants were repaired by donor DNA (5/16; 31%) (Table 2 and Supplemental Fig. 3B and 3 C). The observation that the addition of higher amounts of exogenously added donor DNA results in a lower percentage of transformants repaired with the donor DNA in counterintuitive. It will be of interest to put more effort in this observation (e.g. use an wider range of donor DNA amounts to prove that the observation is reproducible and dependent on the amount of DNA added. It should also be noted that this effect was not observed in the PKV5.4 strain transformed with wither 2 and 12 µg donor (Table 2). Nevertheless, the results clearly and consistently indicate that exogenously added donor DNA is preferred as repair DNA over homologous DNA present on other chromosomes. The *agdB::GLS-K1* is localized on the same chromosomal arm of Chr2 as the *pepB* locus (Fig. 2C). The results indicate a decrease in DNA repair via exogenously added donor DNA in SF11.1, which can either be due to the higher number of copies of potential chromosomal donor DNA or due to the fact that one of the chromosomal donor DNA sequence (*agdB::glaA*_*538*_*-6xHis*) is located on the same chromosomal arm as *pepB*. The *pepB* and *agdB* genes are separated by approximately 2.6 Mb. The higher repair frequencies by chromosomal DNA in the SF11.1 strain compared to PKV5.4 in the presence of donor DNA can be either the result of multiple copies of donor DNA in SF11.1 or can be the result of more efficient intra-chromosomal DNA repair via the *agdB* locus. Since the *agdB* locus contains the *gla*_*538*_*-6xHis* gene, like in the case of the other GLSs, the origin of the DNA which has repaired the *pepB* locus is unknown.

#### Comparison of frequencies of intra-chromosomal versus inter-chromosomal DNA repair after CRISPR/Cas9-induced DSB

Previous work in *S. cerevisiae* has shown that DNA repair of DSBs is generally more efficient if the homologous DNA is on the same chromosome (intra-chromosomal repair) compared to inter-chromosomal events (Agmon et al. 2009; Lee et al. 2016; Wang et al. 2017). To study whether DNA repair of DSBs is generally more efficient if the homologous DNA is on the same chromosome (intra-chromosomal repair) compared to inter-chromosomal repair, *A. niger* strain MA1025.4 was used (Arentshorst et al. 2023). The strain lineage towards MA1025.4 is shown in Fig. 1. The genetic make-up of this strain is schematically shown in Fig. 2D and summarized in Table 1. In this strain 10 glucoamylase landing sites are introduced at 10 different loci. The *pepB* and the *pepN* loci are located close to each other on the right arm of chromosome 2 (Fig. 2D). The distance between the two genes is about 47 kB (https://mycocosm.jgi.doe.gov/Aspni_NRRL3_1/Aspni_NRRL3_1.home.html. In MA1025.4, the *pepN* locus is deleted and replaced with a KORE5 containing GLS (GLS-KORE5), while *pepB* is deleted and replaced with a KORE4 containing GLS (GLS-KORE4). Both the KORE5 and KORE4 sites are unique and not used in the GLSs of the other genes (Fig. 2D; Table 1). In addition, at the telomeric region of chromosome 2, the *goxC* gene is deleted and replaced with a GLS harboring both the KORE1 and KORE3 sequences (Fig. 2D; Table 1).

Hence, MA1025.4 was used to analyze whether intra-chromosomal repair was preferred over inter-chromosomal repair. First, MA1025.4 was transformed with plasmid pFC332-sgKORE4 to create a DSB at the *pepB* locus. In the absence of donor DNA during the transformation, the DSB is expected to be repaired by homologous chromosomal DNA. The number of transformants with pFC332-sgKORE4 was comparable to the transformation efficiency with pFC332 (positive control) indicating efficient repair (Supplemental Fig. 4 and Supplementary Table 3). Nineteen transformants were purified and the DNA repair event at the *pepB* locus was analyzed by amplifying the *pepB* landing sites and subsequent sequencing of the KORE sequence within the GLA landing sites of the amplified PCR fragment. All nineteen transformants amplified a PCR fragment corresponding with the length of a GLS and sequencing of the individual transformants revealed that seventeen transformants were found to contain the KORE5 sequence, and two transformants contained the KORE1 sequence (Table 3 and Supplemental Fig. 5A). The results indicate that the DSB at the *pepB* locus is most efficiently (17/19; 90%) repaired by the neighboring GLS at the *pepN* landing site containing the KORE5 sequence. Repair by the integration of a KORE1 landing site (2/19; 10%) indicates the occurrence of inter-chromosomal repair (Supplemental Fig. 5). Since the KORE1 containing landing sites are not unique, it is not known which landing site was used for repair. The GLS at the *goxC* locus which is, like *pepB* and *pepN*, located on the right arm of chromosome 2 was not used as this GLS contains the KORE1 + KORE3 sequence. This last result suggests that the distance between the *pepB/pepN* loci and *goxC* of 3.5 Mb is too large to allow efficient repair.

**Table 3:** Homologous recombination frequencies in MA1025.4 after Cas9-induced DSB using KORE4 or KORE5 specific guide RNAs for *pepB* and *pepN* loci

Strain	Locus/KORE	Donor DNA*	# of transformants analysed	# of transformants repaired with donor DNA	Chromosomal repair					Donor DNA repair %	Chromosomal repair %	
					K1	K1 + K2	K1 + K3	K4	K5		Close**	Distant**
MA1025.4	*pepB*/KORE4	−	19	−	2	−	−	−	17	−	90%	10%
MA1025.4	*pepB*/KORE4	+	20	8	2	−	−	−	10	40%	50%	10%
MA1025.4	*pepN*/KORE5	−	15	-	1	1	−	13	−	−	87%	13%
MA1025.4	*pepN*/KORE5	+	19	1	1	1	1	15	-	5%	79%	16%

* 2 µg *PglaA-lux-TglaA* PCR fragment. **Close = repaired by the nearby *pepN* locus in the case of the use of KORE4 at the *pepB* locus, or repaired by the nearby *pepB* locus in the case of the use of KORE5 at the *pepN* locus; Distant = repaired by a GLS other that the landing site of *pepB* or *pepN*

In the reciprocal experiment, MA1025.4 was transformed with plasmid pFC332-sgKORE5 to create a DSB at the *pepN* locus. A 70% reduction in the number of transformants was observed for the pFC332-sgKORE5 transformation compared to the transformation efficiency with pFC332 (positive control) (Supplemental Fig. 4). Fifteen transformants obtained when no donor DNA was added were purified and analyzed by PCR. From all fifteen transformants a PCR fragment was amplified corresponding with a GLS (Supplemental Fig. 6). The KORE sequence at the repaired *pepN* locus was analyzed by sequencing. Of the fifteen *pepN l*oci, thirteen were found to contain the KORE4 sequence, one contained the KORE1 sequence and one the KORE1 + 2 sequence (Supplemental Fig. 6A and data not shown). The results indicate that the DSB at the *pepN* locus is most efficiently (13/15; 87%) repaired by the neighboring *pepB* landing site containing the KORE4 sequence. Repair by the integration of a KORE1 landing site (1/15; 6.5%) or a KORE1 + K2 (1/15; 6.5%) indicate also the less-preferred occurrence of inter-chromosomal repair. Since both KORE1 and KORE1 + KORE2-containing landing sites are not unique, it is not known which landing site was used for repair. No repair was found using the g*oxC* locus as KORE1 + KORE3 was not found among the transformants. In conclusion, the results indicate that a DSB is efficiently repaired by a closely localized homologous DNA sequence.

#### Comparison of intra-chromosomal homology-based versus exogenously added donor DNA repair

Strain MA1025.4 was also used to assess whether exogenously added donor DNA is preferred over intra-chromosomal repair. To analyze this, MA1025.4 was transformed with pFC332-sgKORE4 to create a DSB at the *pepB* locus. During transformation, 2 µg *PglaA-lux-TglaA* donor DNA was added. The number of colonies on the transformation plate was comparable to the control (pFC332) and the number of colonies obtained without donor DNA (Supplemental Fig. 4). Twenty transformants were purified and subjected to plasmid loss and by a combination of diagnostic PCR and sequencing, the events of DNA repair were analyzed. Of the twenty transformants, eight transformants showed integration of the donor DNA (8/20 ;40%). Of the twelve remaining transformants ten contained the KORE5 sequence, indicating that the neighboring GLS at the *pepN* locus was used for repair and two transformants contained a KORE1-containg GLS (Table 3 and Supplemental Fig. 5B). In the reciprocal experiment pFC332-sgKORE5 was used to create a DSB at the *pepN* locus. During transformation again 2 µg *PglaA-lux-TglaA* donor DNA was added. The number of transformants was again comparable to the control transformation with pFC332 or the transformation without donor DNA (Supplemental Fig. 4). Diagnostic PCR of nineteen purified transformants that lost the pFC332-sgKORE5 plasmid, revealed that only a single mutant was repaired with exogenously added donor DNA (Table 3 and Supplemental Fig. 6B). Of the remaining eighteen transformants, fifteen transformants were repaired with a GLS containing a KORE4 sequence, indicating repair by the neighboring GLS at the *pepB* locus. One transformant was repaired with the GLS containing KORE1 + 3). One additional transformant was repaired with a KORE1-containing GLS and one transformant was repaired with a KORE1 + 2-containing GLS. The results indicate that there is a locus specific difference in how the DSB is repaired between the two different loci in case of the presence of donor DNA. When donor DNA is present, the *pepN* locus is most efficiently repaired by intra-chromosomal repair (14/19; 74%) and hardly repaired by donor DNA (1/19; 5%). For the *pepB* locus, again most repairs were done via the GLA-KORE5 from the neighboring *pepN* locus (10/20) but also a decent number of transformants were repaired by donor DNA (8/20). The underlying mechanism for the different recombination efficiencies between the two loci is currently unknown. In previous studies it has been shown that homologous recombination is locus specific e.g. for the deletion of the *hdrC* gene in *A. niger* (Carvalho et al. 2011). To the best of our knowledge the reason why homologous recombination frequencies are locus dependent has not been systematically analyzed, but might involve the methylation/acetylation state of the DNA and consequently the level of DNA condensation.

## Conclusions

The iterative method of CRISPR/Cas9-mediated genome editing paved the way to generate *A. niger* strains containing multiple predetermined landing sites to integrate a GOI (Arentshorst et al. 2023). These landing sites were introduced at loci to replace genes encoding proteins with unwanted side effects such as proteases that might cause proteolytic degradation of the recombinant protein, or abundantly present secreted proteins which might hamper in a later phase the separation of the protein of interest from background proteins. Although the method is working well and allowed a stepwise filling of up to all ten landing sites introduced, we noticed in that study as well as in other recent studies (unpublished data) that the efficiency of filling the landing sites was not optimal and that not all landing sites were always filled with the GOI. A possible explanation for that observation is that homologous DNA, present in the genome, acts as donor DNA to repair some of the DSBs created.

The data presented in this study unambiguously show that both inter-chromosomal and intra-chromosomal DNA can act as donor DNA to repair CRISPR/Cas9-induced DSBs. In general, we found that the addition of donor DNA, in our case added during the transformation procedure as double stranded PCR product, was preferred as template to mediate homology directed repair. Multiple copies of homologous DNA sequences and/or potential donor sequences in the proximity to the DSB break reduced the efficiency of incorporation of the GOI. Especially the presence of homologous DNA sequences in close proximity to DSB sites intended to be filled with the gene of interest, should be avoided when designing multicopy production strains.

## Supplementary Information

Supplementary Material 1. Table 1. Primers used in this study.

Supplementary Material 2. Table 2. Number of transformants and relative transformation efficiencies after CRISPR/Cas9-mediated transformation with and without donor DNA.

Supplementary Material 3. Fig. 1. Pictures of the transformation plates of various *A. niger* strains (PKV5.4, SF11.1 and SF29.1) with pFC332 (2 µg) (positive control), pFC332-KORE5 (2 µg), low donor DNA pFC332-KORE5 (2 µg) and 2 µg donor DNA (PglaA-lux-TglaA), high donor DNA pFC332-KORE5 (2 µg) and 12 µg donor DNA ( *PglaA-lux-TglaA *), and negative control (no DNA). Pictures of the plates were taken after five days of growth at 30 °C. n.d. = not determined.

Supplementary Material 4. Fig. 2. Diagnostic PCR results of PKV5.4 transformations. Strain PKV5.4 was transformed with pFC332-KORE5 without the addition of donor DNA (SF30 series), pFC332-KORE5 with 2 µg donor DNA (SF32 series) and pFC332-KORE5 with 12 µg donor DNA (SF33 series). Transformants were purified and subsequently grown on MM without hygromycin to allow plasmid loss. DNA was isolated from the transformants that lost the pFC332 plasmid and used in a diagnostic PCR using *pepB* specific primers. Repair by the endogenous *glaA* locus is expected to yield a PCR product of 3846 bp, repair by the donor DNA fragment ( *PglaA-lux-TglaA* ) is expected to yield a PCR product of 3321 bp. C = control PCR fragment of a *pepB::glaA538*-*6xHis* transformant with an expected size of 3558 bp.

Supplementary Material 5. Fig. 3. Diagnostic PCR results of SF11.1 transformations. Strain SF11.1 was transformed with pFC332-KORE5 without the addition of donor DNA (SF34 series), pFC332-KORE5 with 2 µg donor DNA (SF35 series) and pFC332-KORE5 with 12 µg donor DNA (SF36 series). Transformants were purified and subsequently grown on MM without hygromycin to allow plasmid loss. DNA was isolated from the transformants that lost the pFC332 plasmid and used in a diagnostic PCR using *pepB* specific primers. Repair by the donor DNA fragment (*PglaA-lux-TglaA*) is expected to yield a PCR product of 2951 bp. Repair by the endogenous homologous loci containing the gla- 538 -6xHis is expected to yield a PCR with an expected size of 3558 bp. Black numbering of the transformants indicates DSB repair by *glaA *_538_ -6xHis loci, red numbering of the transformant indicates DSB repair by donor DNA ( *PglaA-lux-TglaA*); blue numbering indicates an inconclusive result.

Supplementary Material 6. Fig. 4. Pictures of the transformation plates of *A. niger* strain MA1025.4 with pFC332 (2 µg) (positive control), pFC332-KORE4 (2 µg), pFC332-KORE4 (2 µg) and 2 µg donor DNA (PglaA-lux-TglaA), and no DNA control (upper row) and pFC332-KORE5 (2 µg), pFC332-KORE5 (2 µg) and 2 µg donor DNA ( *PglaA-lux-TglaA* ) (lower row). Pictures of the plates were taken after five days of growth at 30 °C.

Supplementary Material 7. Fig. 5. Diagnostic PCR results of MA1029.4 transformations at the *pepB* locus. Strain MA1029.4 was transformed with pFC332-KORE4 without the addition of donor DNA (MA1120 series) or with pFC332-KORE4 with 2 µg donor DNA (MA1121 series). Transformants were purified and subsequently grown on MM without hygromycin to allow plasmid loss. DNA was isolated from the transformants that lost the pFC332 plasmid and used in a diagnostic PCR using *pepB* specific primers. The size of the PCR fragment of the *pepB::GLS* locus has an expected size of 1308 bp. Repair by the donor DNA fragment ( *PglaA-lux-TglaA* ) is expected to yield a PCR product of 2951 bp. Each PCR fragment with a size of 938 bp was sequenced to determine the KORE sequence. Black numbering of the transformants indicates that the KORE5 sequence was present, green numbering indicates that the KORE1 sequence was present. Red numbering shows that the donor DNA was integrated at the *pepB* locus. The expected size of the wild type (WT) *pepB* locus is 1400 bp. P = parental strain (MA1025.4). Close = the DSB at the *pepB* locus was repaired by the nearby *pepN* locus; Distant = repaired by a GLS other that the landing site of *pepN* .

Supplementary Material 8. Fig. 6. Diagnostic PCR results of MA1029.4 transformations at the *pepN * locus. Strain MA1029.4 was transformed with pFC332-KORE5 without the addition of donor DNA (MA1122 series) or pFC332-KORE5 with 2 µg donor DNA (MA1123 series). Transformants were purified and subsequently grown on MM without hygromycin to allow plasmid loss. DNA was isolated from the transformants that lost the pFC332 plasmid and used in a diagnostic PCR using *pepN * specific primers. The size of the PCR fragment of the *pepN::GLS* locus has an expected size of 964 bp. Repair by the donor DNA fragment (*PglaA-lux-TglaA*) is expected to yield a PCR product of 2977 bp. Each PCR fragment with a size of 964 bp was sequenced to determine the KORE sequence. Black numbering of the transformants indicates that the KORE4 sequence was present, green numbering indicates that another KORE sequence was presented as indicated. Red numbering shows that the donor DNA was integrated at the *pepN* locus. The expected size of the wild type (WT) *pepN* locus is 2100 bp. P = parental strain (MA1025.4). **Close = the DSB at the *pepN* locus was repaired by the nearby *pepB* locus. Distant = repaired by a GLS other that the landing site of *pepB* .

## Data Availability

No datasets were generated or analysed during the current study.

## References

[CR1] Agmon N, Pur S, Liefshitz B, Kupiec M. Analysis of repair mechanism choice during homologous recombination. Nucleic Acids Res. 2009;37(15):5081–92. 10.1093/nar/gkp495. 19553188 10.1093/nar/gkp495 PMC2731894

[CR2] Aleksenko A, Clutterbuck AJ. The plasmid replicator AMA1 in *aspergillus nidulans* is an inverted duplication of a low-copy-number dispersed genomic repeat. Mol Microbiol. 1996;19(3):565–74. 10.1046/j.1365-2958.1996.400937.x. 8830247 10.1046/j.1365-2958.1996.400937.x

[CR3] Arentshorst M, Ram AFJ, Meyer V. Using non-homologous end-joining-deficient strains for functional gene analyses in filamentous fungi. Methods Mol Biol. 2012;835:133–50. 10.1007/978-1-61779-501-5_9. 22183652 10.1007/978-1-61779-501-5_9

[CR4] Arentshorst M, Lagendijk EL, Ram AFJ. A new vector for efficient gene targeting to the *pyrG* locus in *Aspergillus Niger*. Fungal Biol Biotechnol. 2015;14:2–2. 10.1186/s40694-015-0012-4. 10.1186/s40694-015-0012-4 PMC5611571 28955454

[CR5] Arentshorst M, Falco MD, Moisan MC, Reid ID, Spaapen TOM, van Dam J, Demirci E, Powlowski J, Punt PJ, Tsang A, Ram AFJ. Identification of a conserved transcriptional activator-repressor module controlling the expression of genes involved in tannic acid degradation and gallic acid utilization in *Aspergillus Niger*. Front Fungal Biol. 2021;2:681631. 10.3389/ffunb.2021.681631. 37744122 10.3389/ffunb.2021.681631 PMC10512348

[CR6] Arentshorst M, Kooloth Valappil P, Mózsik L, Regensburg-Tuïnk TJG, Seekles SJ, Tjallinks G, Fraaije MW, Visser J, Ram AFJ. A CRISPR/Cas9-based multicopy integration system for protein production in *Aspergillus Niger*. FEBS J. 2023. 10.1111/febs.16891. 37335926 10.1111/febs.16891

[CR7] Behera BC. Citric acid from *Aspergillus Niger*: a comprehensive overview. Crit Rev Microbiol. 2020;46(6):727–49. 10.1080/1040841X.2020.1828815. 33044884 10.1080/1040841X.2020.1828815

[CR8] Bos CJ, Debets AJM, Swart K, Huybers A, Kobus G, Slakhorst SM. Genetic analysis and the construction of master strains for assignment of genes to six linkage groups in *Aspergillus Niger*. Curr Genet. 1988;14:437–43. 3224384 10.1007/BF00521266

[CR9] Cairns TC, Nai C, Meyer V. How a fungus shapes biotechnology: 100 years of *Aspergillus Niger* research. Fungal Biol Biotechnol. 2018;5:13. 10.1186/s40694-018-0054-5. 29850025 10.1186/s40694-018-0054-5 PMC5966904

[CR10] Cairns TC, Barthel L, Meyer V. Something old, something new: challenges and developments in *Aspergillus Niger* biotechnology. Essays Biochem. 2021;65(2):213–24. 10.1042/EBC20200139. 33955461 10.1042/EBC20200139 PMC8314004

[CR11] Carvalho ND, Arentshorst M, Jin Kwon M, Meyer V, Ram AFJ. Expanding the *ku70* toolbox for filamentous fungi: establishment of complementation vectors and recipient strains for advanced gene analyses. Appl Microbiol Biotechnol. 2010;87(4):1463–73. 10.1007/s00253-010-2588-1. Epub 2010 Apr 27. 20422182 10.1007/s00253-010-2588-1 PMC2892608

[CR12] Carvalho ND, Arentshorst M, Kooistra R, Stam H, Sagt CM, van den Hondel CA, Ram AFJ. Effects of a defective ERAD pathway on growth and heterologous protein production in *Aspergillus niger*. Appl Microbiol Biotechnol. 2011;89(2):357–73. 10.1007/s00253-010-2916-5. 20922374 10.1007/s00253-010-2916-5 PMC3016150

[CR13] Demirci E, Arentshorst M, Yilmaz B, Swinkels A, Reid ID, Visser J, Tsang A, Ram AFJ. Genetic characterization of mutations related to conidiophore stalk length development in *aspergillus Niger* Laboratory strain N402. Front Genet. 2021;12:666684. 10.3389/fgene.2021.666684. 33959152 10.3389/fgene.2021.666684 PMC8093798

[CR14] Garneau JE, Dupuis MÈ, Villion M, Romero DA, Barrangou R, Boyaval P, Fremaux C, Horvath P, Magadán AH, Moineau S. The CRISPR/Cas bacterial immune system cleaves bacteriophage and plasmid DNA. Nature. 2010;468(7320):67–71. 10.1038/nature0952. 21048762 10.1038/nature09523

[CR15] Garrigues S, Peng M, Kun RS, de Vries RP. Non-homologous end-joining-deficient filamentous fungal strains mitigate the impact of off-target mutations during the application of CRISPR/Cas9. mBio. 2023;14(4):e0066823. 10.1128/mbio.00668-23. 37486124 10.1128/mbio.00668-23 PMC10470509

[CR16] Jin FJ, Wang BT, Wang ZD, Jin L, Han P. CRISPR/Cas9-based genome editing and its application in aspergillus species. J Fungi (Basel). 2022;8(5):467. 10.3390/jof8050467. 35628723 10.3390/jof8050467 PMC9143064

[CR17] Kun RS, Meng J, Salazar-Cerezo S, Mäkelä MR, de Vries RP, Garrigues S. CRISPR/Cas9 facilitates rapid generation of constitutive forms of transcription factors in Aspergillus Niger through specific on-site genomic mutations resulting in increased saccharification of plant biomass. Enzyme Microb Technol. 2020;136:109508 10.1016/j.enzmictec.2020.1095. 32331715 10.1016/j.enzmictec.2020.109508

[CR18] Labun K, Montague TG, Gagnon JA, Thyme SB, Valen E. CHOPCHOP v2: a web tool for the next generation of CRISPR genome engineering. Nucleic Acids Res. 2016. 10.1093/nar/gkw39. 27185894 10.1093/nar/gkw398 PMC4987937

[CR19] Lee CS, Wang RW, Chang HH, Capurso D, Segal MR, Haber JE. Chromosome position determines the success of double-strand break repair. Proc Natl Acad Sci USA. 2016;113(2):E146–54. 10.1073/pnas.1523660113. 26715752 10.1073/pnas.1523660113 PMC4720327

[CR20] Li C, Zhou J, Du G, Chen J, Takahashi S, Liu S. Developing *aspergillus Niger* as a cell factory for food enzyme production. Biotechnol Adv. 2020;44:107630. 10.1016/j.biotechadv.2020.107630. 32919011 10.1016/j.biotechadv.2020.107630

[CR21] Meyer V, Arentshorst M, El-Ghezal A, Drews AC, Kooistra R, van den Hondel CA, Ram AFJ. Highly efficient gene targeting in the *aspergillus Niger kusA* mutant. J Biotechnol. 2007;128(4):770–5. 10.1016/j.jbiotec.2006.12.021. 17275117 10.1016/j.jbiotec.2006.12.021

[CR22] Nødvig CS, Nielsen JB, Kogle ME, Mortensen UH. A CRISPR-Cas9 system for genetic engineering of filamentous fungi. PLoS ONE. 2015;10(7):e0133085. 10.1371/journal.pone.0133085. 26177455 10.1371/journal.pone.0133085 PMC4503723

[CR23] Sander JD, Joung JK. CRISPR-Cas systems for editing, regulating and targeting genomes. Nat Biotechnol. 2014;32(4):347–55. 10.1038/nbt.2842. Epub 2014 Ma. 24584096 10.1038/nbt.2842 PMC4022601

[CR24] Seekles SJ, van den Brule T, Punt M, Dijksterhuis J, Arentshorst M, Ijadpanahsaravi M, Roseboom W, Meuken G, Ongenae V, Zwerus J, Ohm RA, Kramer G, Wösten HAB, de Winde JH, Ram AFJ. Compatible solutes determine the heat resistance of conidia. Fungal Biol Biotechnol. 2023;10(1):21. 10.1186/s40694-023-00168-9. 37957766 10.1186/s40694-023-00168-9 PMC10644514

[CR25] Song L, Ouedraogo JP, Kolbusz M, Nguyen TTM, Tsang A. Efficient genome editing using tRNA promoter-driven CRISPR/Cas9 gRNA in *Aspergillus Niger*. PLoS ONE. 2018;13(8):e0202868. 10.1371/journal.pone.0202868. 30142205 10.1371/journal.pone.0202868 PMC6108506

[CR26] van Leeuwe TM, Arentshorst M, Ernst T, Alazi E, Punt PJ, Ram AFJ. Efficient marker free CRISPR/Cas9 genome editing for functional analysis of gene families in filamentous fungi. Fungal Biol Biotechnol. 2019;6:13. 10.1186/s40694-019-0076-7. 31559019 10.1186/s40694-019-0076-7 PMC6754632

[CR27] Wang RW, Lee CS, Haber JE. Position effects influencing intrachromosomal repair of a double-strand break in budding yeast. PLoS ONE. 2017;12(7):e0180994. 10.1371/journal.pone.0180994. 28700723 10.1371/journal.pone.0180994 PMC5507452

[CR28] Yu R, Liu J, Wang Y, Wang H, Zhang H. *Aspergillus**niger* as a secondary metabolite factory. Front Chem. 2021;30:9:701022. 10.3389/fchem.2021.701022. 10.3389/fchem.2021.701022 PMC8362661 34395379

