# Supplementary material for: Competition between homologous chromosomal DNA and exogenous donor DNA to repair CRISPR/Cas9-induced double-strand breaks in Aspergillus niger
Source: Fungal Biol Biotechnol. 2024 Oct 15;11:15. doi: 10.1186/s40694-024-00184-3 (PMC11481784; doi:10.1186/s40694-024-00184-3)
Supplement: Supplementary file 2 — Supplementary Material 2. Table 2. Number of transformants and relative transformation efficiencies after CRISPR/Cas9-mediated transformation with and without donor DNA. [file 40694_2024_184_MOESM2_ESM.docx]

Supplemental Table 2. Number of transformants and relative transformation efficiencies after CRISPR/Cas9-mediated transformation with and without donor DNA

| Strain | pFC332 |  | KORE 5 Guide  (no donor) |  | 2 µg  donor | |  | 12 µg  donor | | negative | | % colonies pFC332 vs KORE5 | | % colonies pFC332 vs  2µg DNA donor | | % colonies pFC332 vs  12µg DNA donor | |
| --- | --- | --- | --- | --- | --- | --- | --- | --- | --- | --- | --- | --- | --- | --- | --- | --- | --- |
|  | 404 |  | 35 |  | 198 | |  | 191 | | 0 | | 8.7 | | 49.0 | | 47.3 | |
| PKV5.4 | 306 |  | 28 |  | 139 | |  | 257 | | 0 | | 9.2 | | 45.4 | | 84.0 | |
|  | 269 |  | 87 |  | n.d.* | |  | n.d.* | | 0 | | 32.3 | | n.d.* | | n.d.* | |
|  | 326 ± 57 |  | 50 ± 26 |  | 169 ± 30 | |  | 224 ± 33 | | 0 | | 16.7 ± 11.0 | | 47.2 ± 1.8 | | 65.6 ± 18.4 | |
|  |  |  |  |  |  | |  |  | |  | |  | |  | |  | |
|  | 193 |  | 35 |  | 302 | |  | 155 | | 0 | | 17.9 | | 154.9 | | 79.5 | |
| SF11.1 | 195 |  | 20 |  | 131 | |  | 101 | | 0 | | 10.4 | | 67.9 | | 52.3 | |
|  | 326 |  | 64 |  | 362 | |  | 108 | | 0 | | 19.6 | | 111.0 | | 33.1 | |
|  | 238 ± 62 |  | 40 ± 18 |  | 265 ± 98 | |  | 121 ± 24 | | 0 | | 16.0 ± 4.0 | | 111.3 ± 35.5 | | 55.0 ± 19.0 | |
|  |  |  |  |  |  | |  |  | |  | |  | |  | |  | |
| SF29.1 | 280 |  | 1 |  | 600 | |  | n.d.* | | 0 | | 0.36 | | 214.3 | | n.d.* | |
|  |  |  |  |  |  |  |  | |  | |  | |  | |  | |  |

* n.d. – not determined.
