# Supplementary figures and images for: Competition between homologous chromosomal DNA and exogenous donor DNA to repair CRISPR/Cas9-induced double-strand breaks in Aspergillus niger
Source: Fungal Biol Biotechnol. 2024 Oct 15;11:15. doi: 10.1186/s40694-024-00184-3 (PMC11481784; doi:10.1186/s40694-024-00184-3)

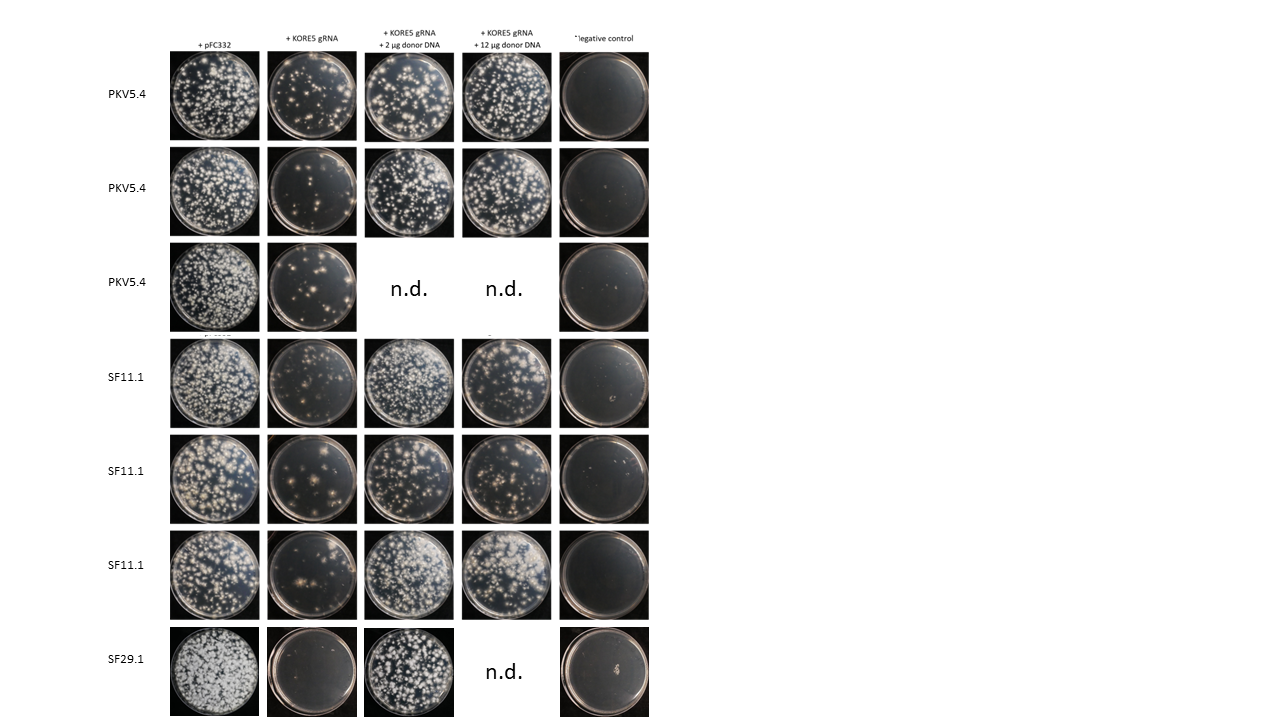

Supplement: Supplementary file 3 — Supplementary Material 3. Fig. 1. Pictures of the transformation plates of various A. niger strains (PKV5.4, SF11.1 and SF29.1) with pFC332 (2 µg) (positive control), pFC332-KORE5 (2 µg), low donor DNA pFC332-KORE5 (2 µg) and 2 µg donor DNA (PglaA-lux-TglaA), high donor DNA pFC332-KORE5 (2 µg) and 12 µg donor DNA ( PglaA-lux-TglaA ), and negative control (no DNA). Pictures of the plates were taken after five days of growth at 30 °C. n.d. = not determined. [file 40694_2024_184_MOESM3_ESM.tif]

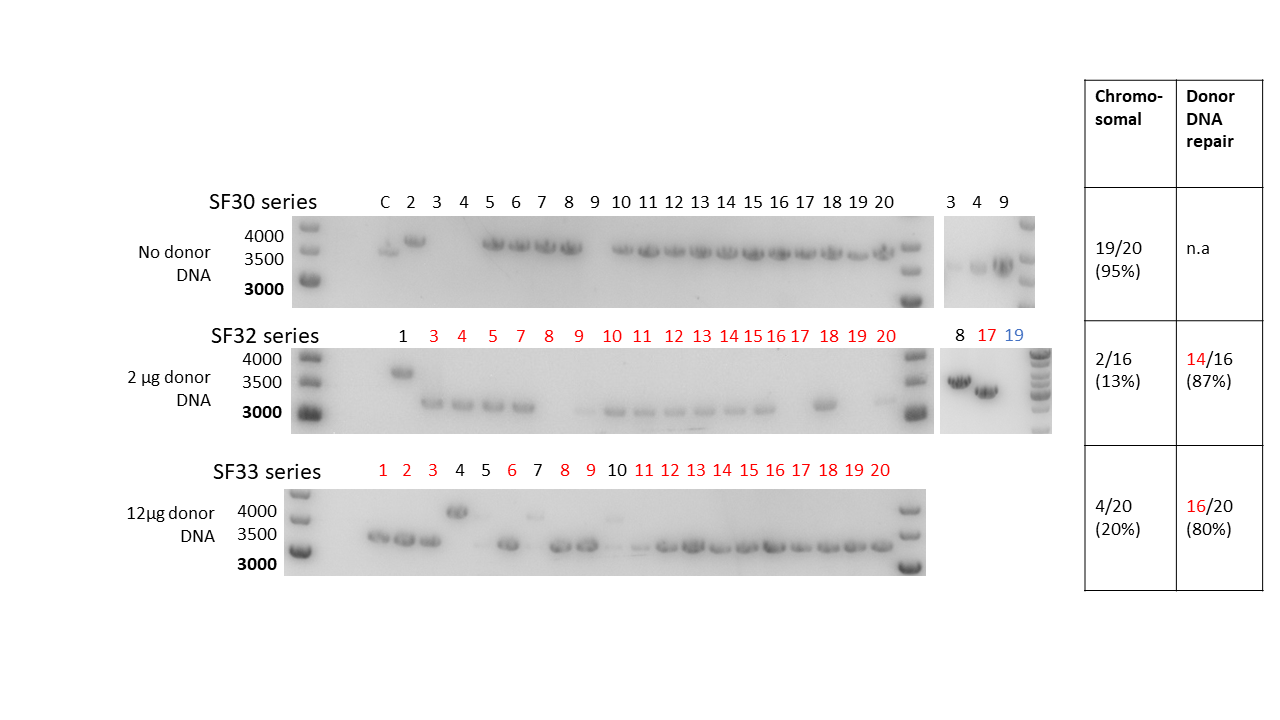

Supplement: Supplementary file 4 — Supplementary Material 4. Fig. 2. Diagnostic PCR results of PKV5.4 transformations. Strain PKV5.4 was transformed with pFC332-KORE5 without the addition of donor DNA (SF30 series), pFC332-KORE5 with 2 µg donor DNA (SF32 series) and pFC332-KORE5 with 12 µg donor DNA (SF33 series). Transformants were purified and subsequently grown on MM without hygromycin to allow plasmid loss. DNA was isolated from the transformants that lost the pFC332 plasmid and used in a diagnostic PCR using pepB specific primers. Repair by the endogenous glaA locus is expected to yield a PCR product of 3846 bp, repair by the donor DNA fragment ( PglaA-lux-TglaA ) is expected to yield a PCR product of 3321 bp. C = control PCR fragment of a pepB::glaA538-6xHis transformant with an expected size of 3558 bp. [file 40694_2024_184_MOESM4_ESM.tif]

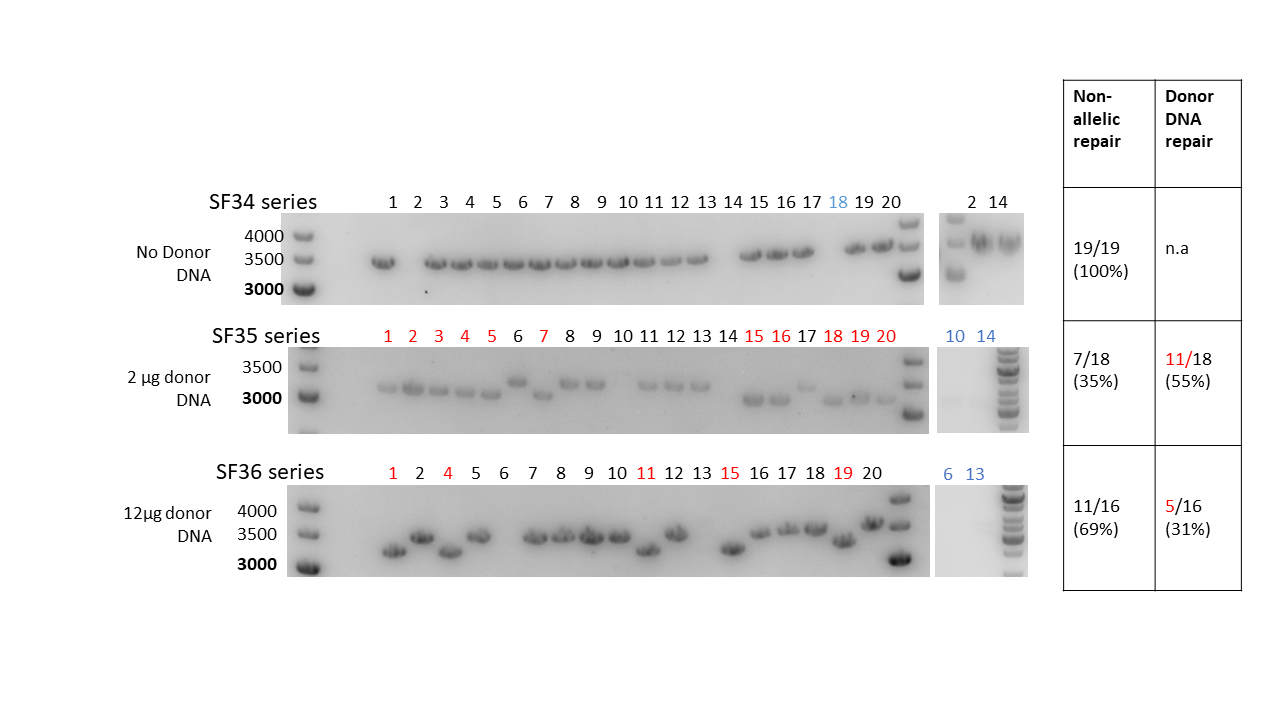

Supplement: Supplementary file 5 — Supplementary Material 5. Fig. 3. Diagnostic PCR results of SF11.1 transformations. Strain SF11.1 was transformed with pFC332-KORE5 without the addition of donor DNA (SF34 series), pFC332-KORE5 with 2 µg donor DNA (SF35 series) and pFC332-KORE5 with 12 µg donor DNA (SF36 series). Transformants were purified and subsequently grown on MM without hygromycin to allow plasmid loss. DNA was isolated from the transformants that lost the pFC332 plasmid and used in a diagnostic PCR using pepB specific primers. Repair by the donor DNA fragment (PglaA-lux-TglaA) is expected to yield a PCR product of 2951 bp. Repair by the endogenous homologous loci containing the gla- 538 -6xHis is expected to yield a PCR with an expected size of 3558 bp. Black numbering of the transformants indicates DSB repair by glaA 538 -6xHis loci, red numbering of the transformant indicates DSB repair by donor DNA ( PglaA-lux-TglaA); blue numbering indicates an inconclusive result. [file 40694_2024_184_MOESM5_ESM.tif]

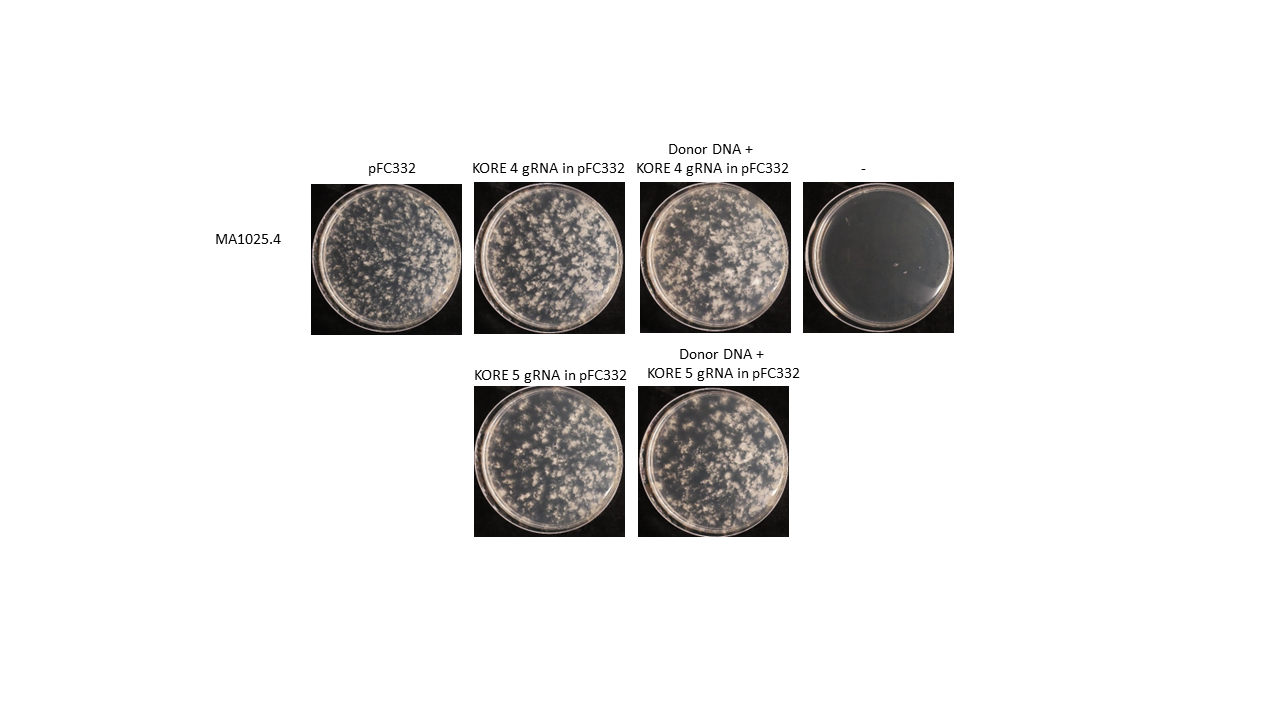

Supplement: Supplementary file 6 — Supplementary Material 6. Fig. 4. Pictures of the transformation plates of A. niger strain MA1025.4 with pFC332 (2 µg) (positive control), pFC332-KORE4 (2 µg), pFC332-KORE4 (2 µg) and 2 µg donor DNA (PglaA-lux-TglaA), and no DNA control (upper row) and pFC332-KORE5 (2 µg), pFC332-KORE5 (2 µg) and 2 µg donor DNA ( PglaA-lux-TglaA ) (lower row). Pictures of the plates were taken after five days of growth at 30 °C. [file 40694_2024_184_MOESM6_ESM.tif]

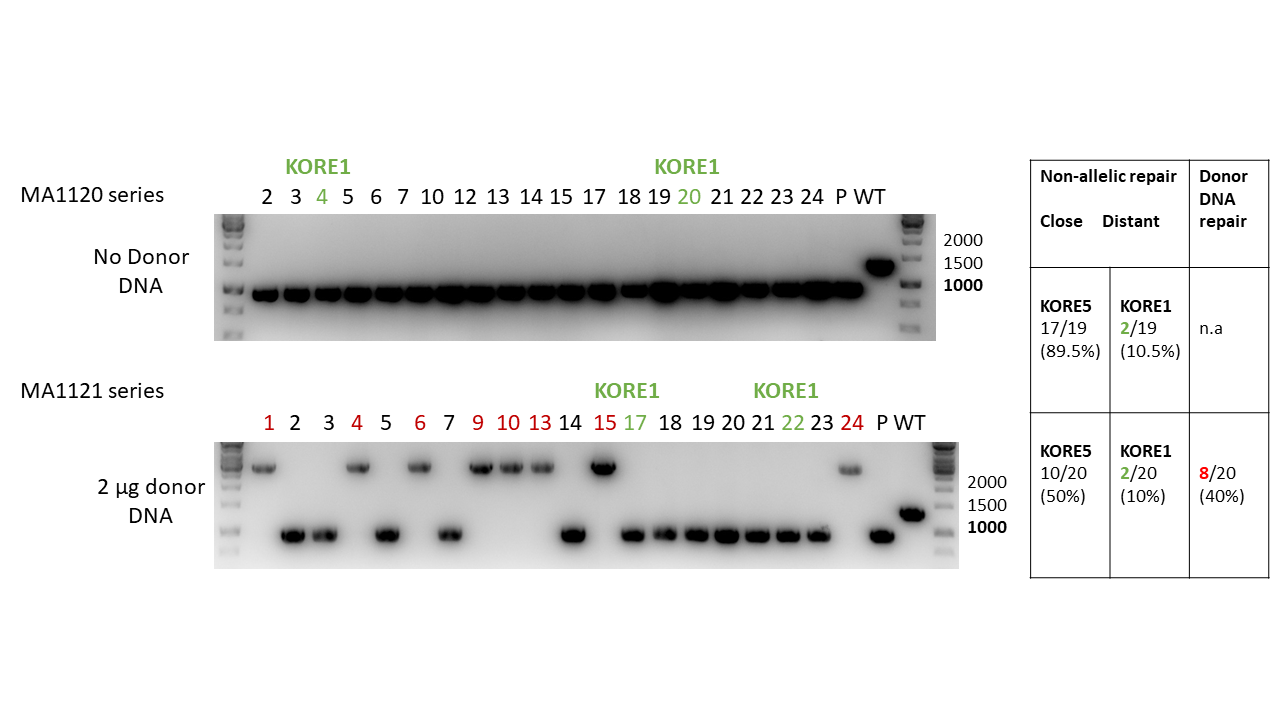

Supplement: Supplementary file 7 — Supplementary Material 7. Fig. 5. Diagnostic PCR results of MA1029.4 transformations at the pepB locus. Strain MA1029.4 was transformed with pFC332-KORE4 without the addition of donor DNA (MA1120 series) or with pFC332-KORE4 with 2 µg donor DNA (MA1121 series). Transformants were purified and subsequently grown on MM without hygromycin to allow plasmid loss. DNA was isolated from the transformants that lost the pFC332 plasmid and used in a diagnostic PCR using pepB specific primers. The size of the PCR fragment of the pepB::GLS locus has an expected size of 1308 bp. Repair by the donor DNA fragment ( PglaA-lux-TglaA ) is expected to yield a PCR product of 2951 bp. Each PCR fragment with a size of 938 bp was sequenced to determine the KORE sequence. Black numbering of the transformants indicates that the KORE5 sequence was present, green numbering indicates that the KORE1 sequence was present. Red numbering shows that the donor DNA was integrated at the pepB locus. The expected size of the wild type (WT) pepB locus is 1400 bp. P = parental strain (MA1025.4). Close = the DSB at the pepB locus was repaired by the nearby pepN locus; Distant = repaired by a GLS other that the landing site of pepN . [file 40694_2024_184_MOESM7_ESM.tif]

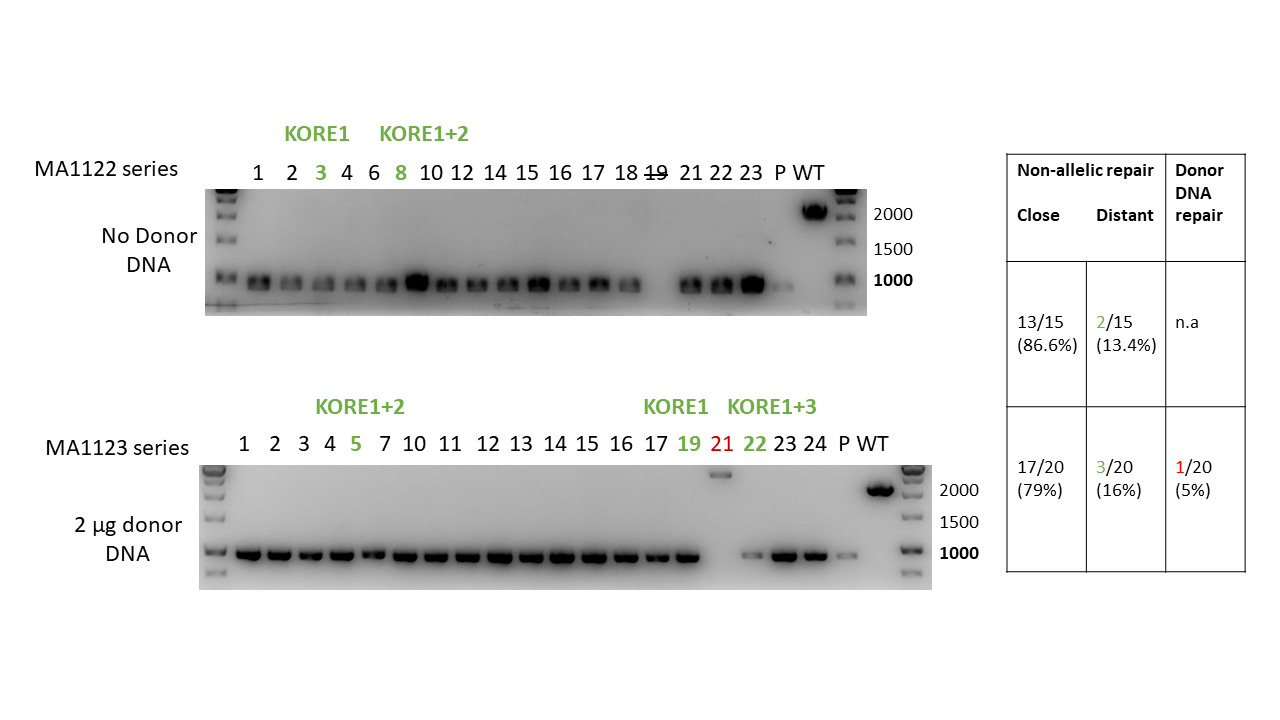

Supplement: Supplementary file 8 — Supplementary Material 8. Fig. 6. Diagnostic PCR results of MA1029.4 transformations at the pepN locus. Strain MA1029.4 was transformed with pFC332-KORE5 without the addition of donor DNA (MA1122 series) or pFC332-KORE5 with 2 µg donor DNA (MA1123 series). Transformants were purified and subsequently grown on MM without hygromycin to allow plasmid loss. DNA was isolated from the transformants that lost the pFC332 plasmid and used in a diagnostic PCR using pepN specific primers. The size of the PCR fragment of the pepN::GLS locus has an expected size of 964 bp. Repair by the donor DNA fragment (PglaA-lux-TglaA) is expected to yield a PCR product of 2977 bp. Each PCR fragment with a size of 964 bp was sequenced to determine the KORE sequence. Black numbering of the transformants indicates that the KORE4 sequence was present, green numbering indicates that another KORE sequence was presented as indicated. Red numbering shows that the donor DNA was integrated at the pepN locus. The expected size of the wild type (WT) pepN locus is 2100 bp. P = parental strain (MA1025.4). **Close = the DSB at the pepN locus was repaired by the nearby pepB locus. Distant = repaired by a GLS other that the landing site of pepB . [file 40694_2024_184_MOESM8_ESM.tif]
